# The Influence of task Demands, Verbal Ability and Executive Functions on Item and Source Memory in Autism Spectrum Disorder

**DOI:** 10.1007/s10803-017-3299-6

**Published:** 2017-09-18

**Authors:** Sara Semino, Melanie Ring, Dermot M. Bowler, Sebastian B. Gaigg

**Affiliations:** 1 Department of Educational Sciences, University of Genoa, Corso A. Podesta, 2, 16128 Genoa, Italy; 2 Department of Child & Adolescent Psychiatry, Medical Faculty of the Technical University Dresden, Dresden, Germany; 3 Autism Research Group, Department of Psychology, City, University of London, Northampton Square, London, EC1V 0HB UK

**Keywords:** Autism, Source memory, Item memory, Recollection, Verbal ability, Executive function

## Abstract

Autism Spectrum Disorder (ASD) is generally associated with difficulties in contextual source memory but not single item memory. There are surprising inconsistencies in the literature, however, that the current study seeks to address by examining item and source memory in age and ability matched groups of 22 ASD and 21 comparison adults. Results show that group differences in source memory are moderated by task demands but not by individual differences in verbal ability, executive function or item memory. By contrast, unexpected group differences in item memory could largely be explained by individual differences in source memory. These observations shed light on the factors underlying inconsistent findings in the memory literature in ASD, which has important implications for theory and practice.

## Introduction

Since the pioneering work of Hermelin and O’Connor in the 1960s and 70 s (Hermelin and O’Connor 1967, 1975) it has been known that Autism Spectrum Disorder (ASD) is associated with a particular pattern of strengths and weaknesses in the domain of learning and memory. A number of complementary explanations of this pattern implicate the encoding and retrieval of contextually rich memories as a source of difficulty for individuals with ASD whilst single item memory (i.e., memory for objects, words, sounds, etc., without necessarily any memory of where, how or when they were studied) is spared. There are surprising inconsistencies in studies of source memory, however, which specifically examine the ability to remember details about the context in which items were presented. The aim of the current study is to shed light on the reasons for this mixed pattern of results, which is important not only for further specifying the mechanisms underlying memory difficulties in ASD but also for informing strategies on how to alleviate them.

The memory profile associated with ASD has recently been the subject of a number of comprehensive reviews (Boucher et al. 2012; Bowler et al. 2011; Gaigg and Bowler 2012). Briefly, procedural and implicit memory, which influence behaviour outside conscious awareness, are generally preserved (Bowler et al. 1997; Gardiner et al. 2003; Nemeth et al. 2010; Ring et al. 2015; Travers et al. 2010). Declarative memory, on the other hand, demonstrates a relatively consistent pattern of strengths and weaknesses, whereby greater difficulties are evident on tests of free recall compared to tests of cued recall or recognition. Free recall requires the unaided retrieval of studied material, and difficulties on such tests are particularly evident in ASD when to-be-remembered stimuli lend themselves to be organised meaningfully into categories or conceptual clusters (Bowler et al. 2010; Gaigg et al. 2008; Loth et al. 2011; Minshew and Goldstein 1993; Smith et al. 2007; Sumiyoshi et al. 2011; Tager-Flusberg 1991; see also; Begeer et al. 2014), or when recall instructions require the re-creation of the specific temporal order or spatial relations of the stimuli (Bowler et al. 2004, 2016; Gaigg et al. 2013; Lind et al. 2013; Poirier et al. 2011). Moreover, when memory for complex events is examined, such as naturalistic videos (Maras and Bowler 2010, 2012b; Maras et al. 2012; McCrory et al. 2007) or personal experiences (Crane and Goddard 2008; Crane et al. 2013; Lind et al. 2014; Maras et al. 2013), free recall is found to be less rich in contextual detail in individuals with ASD, which all suggests difficulties with the organisation of information during initial encoding and/or retrieval.

In contrast to free recall, cued recall and recognition memory pose fewer difficulties for individuals with ASD, although there are important nuances. Specifically, retrieval cues in the form of category labels (‘*Which fruit did you see?’*) are consistently effective in facilitating the recall of lists of words or pictures in ASD (Boucher and Warrington 1976; Bowler et al. 2009, 1997; Mottron et al. 2001; Tager-Flusberg 1991). However, on paired associate cued recall tests, where participants need to remember pairs of items (e.g., Card—Ball) before retrieving one in response to the other (‘*Which word went with ‘Card’?*), the evidence is more mixed, with several studies documenting preserved performance (Ambery et al. 2006; Boucher and Warrington 1976; Gardiner et al. 2003; Minshew and Goldstein 2001; Williams et al. 2005) whilst others document impairments (Bigham et al. 2010 1; Brown et al. 2010; Morton-Evans and Hensley 1978). This suggests that cues that help to organise the retrieval of a number of items tend to be more effective for individuals with ASD than cues that aid the retrieval of specific item–item associations. Studies of recognition memory echo this pattern by showing that the ability to discriminate studied from new objects is generally preserved whereas the ability to place objects back into the screen locations in which they were studied (Ring et al. 2015), or the ability to detect transpositions of object locations or object colours within complex scenes (Bowler et al. 2014; Cooper et al. 2015) is not. In other words, similar to cued-recall tests, recognition tests tend to show preserved memory for single items but compromised memory for specific associations between items and their contexts. This pattern is further supported by the observation that individuals with ASD are less likely than comparison groups to recollect contextual details about items they recognise (Bowler et al. 2007, 2000; Cooper et al. 2015, 2017; Gaigg et al. 2015; Meyer et al. 2014).

As this brief overview illustrates, individuals on the autism spectrum tend to experience disproportionate difficulties in spontaneous free recall, particularly the recall of contextual details about the study episode. A number of complementary explanations have been offered for this pattern. For instance, the *complex information processing* model (CIP; Minshew and Goldstein 1998, 2001) and *Task Support Hypothesis* (TSH; Bowler et al. 2004, 1997) both argue that the wider cognitive phenotype associated with ASD, including in the domain of memory, is a reflection of abnormalities in the integration, organisation and flexible use of information. As a result, the use of organisational strategies (e.g., category clustering) that typically facilitate free recall is attenuated (Bowler et al. 2008, 2009, 2010; Gaigg et al. 2008; Minshew and Goldstein 1993; Sumiyoshi et al. 2011), whilst performance on cued recall or recognition memory tasks that either scaffold or rely less on effective organisational strategies tends to be spared (Bowler et al. 2008a, 2015, 1997; Mottron et al. 2001; Salmond et al. 2005; Toichi and Kamio 2002). Consistent with this view, some studies have observed correlations between memory difficulties and measures of executive functions in ASD (Bennetto et al. 1996; Goddard et al. 2014; Maister et al. 2013) although in some studies these correlations are only weak (e.g., Bowler et al. 2014; O’Shea et al. 2005).

Other explanations for the memory profile in ASD have revolved more around specific memory processes rather than executive functions. For example, one view holds that ASD is characterised by relatively specific impairments in the encoding of *relational* but not *item-specific* information (Bowler et al. 2011; Gaigg et al. 2015, 2008). Relational processes flexibly bind elements of experiences into contextually rich representations of the past whilst *item-specific* processes support the encoding of the individual elements per se (Hunt and Einstein 1981). Since relational processes sub-serve organisational strategies that are important for free recall, and item-specific processes serve a discriminative function that is important for recognition memory (Hunt and Einstein 1981), this distinction accounts for the differential pattern of performance across test procedures in ASD. Moreover, the distinction explains why even on supported test procedures, individuals with ASD experience difficulties retrieving specific item–item and item–context associations (Bowler et al. 2014; Cooper et al. 2015; Ring et al. 2016). A related view argues that the memory profile in ASD reflects difficulties with the process of *recollection*, which describes the spontaneous retrieval of contextually rich representations of prior experiences and contrasts retrieval in the form of *familiarity* that is relatively void of contextual detail (Montaldi and Mayes 2010; Yonelinas 2002). Clearly, this distinction also effectively captures the pattern of strengths and difficulties individuals with ASD experience across memory tasks and substantial evidence confirms that recollection but not familiarity is compromised in ASD (Bowler et al. 2007, 2000; Massand and Bowler 2015; Massand et al. 2013; Meyer et al. 2014; Souchay et al. 2013; Tanweer et al. 2010), at least in individuals who do not have concomitant language and/or intellectual impairments (see Bigham et al. 2010; Ni Chuileann and Quigley 2013).

The explanations that have been offered for the memory profile associated with ASD all concur that the retrieval of contextual information should pose relatively consistent difficulties. Yet on source memory paradigms that specifically probe such retrieval the evidence is surprisingly mixed. In a typical source memory experiment, participants are usually asked to learn a series of unrelated items, such as words or pictures, that are studied under different conditions; the stimuli might be presented by different people, in different locations on a screen or at distinct times. During test, participants are then asked to retrieve the studied items and the specific conditions under which they were studied. Johnson et al. (1993) distinguish between three types of source information: (1) *Internal* information about the operations the participant performed on to-be-remembered items (e.g., whether they said a word out loud or read it quietly), (2) *External* information about *where, when* and *how* the to-be-remembered items were presented (e.g., on the top or bottom of a screen or in a male or female voice) and (3) *Self versus Other* information about whether the participant or someone else acted on to-be-remembered items (e.g., Did the participant or the experimenter read a word or act on an object?). Of the studies that have examined self-other source memory in ASD to date, one has reported no impairment (Williams and Happé 2009), some have reported modest (Cohen’s *d* < 0.3) impairments (Cooper et al. 2016; Farrant et al. 1998; Lind and Bowler 2009), and some have reported more substantial (Cohen’s *d* > 0.6) impairments (Hala et al. 2005; Hill and Russell 2002; Russell and Jarrold 1999) although in the studies by Farrant et al. (1998) and Hill and Russell (2002) the groups of children with ASD demonstrated poorer self-other source memory only vis-a-vis a group of mental age matched younger typically developing (TD) children but not vis-a-vis a group of children with non-specific learning disabilities. Studies examining internal and external source memory are equally inconsistent with some studies reporting difficulties in ASD (Bennetto et al. 1996; Bigham et al. 2010; Hala et al. 2005; O’Shea et al. 2005) whilst others report preserved or only moderately compromised performance (Grainger et al. 2016; Ring et al. 2015; Wojcik et al. 2013).

A number of factors might contribute to the mixed pattern of findings in the source memory literature of ASD. In line with the Task Support Hypothesis (TSH) outlined earlier, Bowler and colleagues have previously shown that individuals with ASD experience far greater source memory difficulties on tests of source recall (*How was the word presented?)* than source recognition (*Did you see the word on the top or bottom of the screen or hear it in a male or female voice;* Bowler et al. 2004, 2015), which suggests that certain task parameters play an important role in moderating source memory difficulties in ASD. However, there remain some source recognition studies that demonstrate performance decrements in ASD (O’Shea et al. 2005) and some source recall studies that show none (Ring et al. 2015), suggesting that other task parameters may also be important. The current study sought to contribute to the literature by systematically manipulating the number of to-be-remembered items and the number of to-be-remembered source locations in which they were studied. The prediction was that source memory would be impaired in ASD vis-a-vis a comparison group overall whilst item memory for the individual objects would be spared. In addition we anticipated that increasing the number of source locations would have more detrimental effects on source memory in ASD than in comparison participants because of the increasing demands of associating items with their specific source locations. By contrast, increasing the number of items was expected to lead to similar effects in both groups under the assumption that this would primarily increase demands on item memory. Finally, we also had the opportunity to examine the role of individual differences in verbal ability and executive functions, which previous studies have found to be associated with source memory difficulties in ASD (Bennetto et al. 1996; Hala et al. 2005; Lind and Bowler 2009, but see; O’Shea et al. 2005).

## Methods

### Participants

Twenty-four ASD and 23 TD individuals took part in this study. They were an opportunity sample recruited from a participant database in the host laboratory with the constraint that groups would be matched on gender, chronological age and Verbal (VIQ), Performance (PIQ), and Full-scale IQ (FIQ) as measured by the third edition of the Wechsler Adult Intelligence Scale (WAIS-III^UK^; The Psychological Corporation, 2000). The WAIS had been administered by a member of the research team on a previous occasion. Two ASD and 2 TD participants were subsequently excluded from further analyses because they did not meet the inclusion criteria described below. The remaining groups, however, continued to be closely matched. The inclusion criteria for the ASD group were that they had been diagnosed by experienced clinicians through the UK’s National Health Service, and that they met clinical cut-off criteria on either one or both of the Autism-Spectrum Quotient (AQ; Baron-Cohen et al. 2001) and Autism Diagnostic Observation Schedule (ADOS; Lord et al. 2000), which was administered by a research reliable member of the research team. Inclusion criteria for the TD group were that they scored below the cut-off of 26 on the AQ (Woodbury-Smith et al. 2005) and that they reported no personal or family history of psychiatric or neurodevelopmental disorders. All except one individual with ASD were native English speakers. Participants were reimbursed for their time with standard university fees and the procedures outlined below were approved by the host Department’s ethics committee in line with the standards set out by the British Psychological Society and the declaration of Helsinki. Summary descriptive statistics for the ASD and TD groups are provided in Table 1.

**Table 1:** Participant characteristics for the ASD and TD groups

Measure	TD (17 m, 4 f)			ASD (18 m, 4f)			*t*(41)	*p*	Cohen’s *d*
	M	SD	Range	M	SD	Range			
Age (years)	44.9	11.4	27.3–61.6	42.5	11.7	26.5–62.4	0.70	0.49	0.21
VIQ^a^	106.0	14.9	76–131	104.8	15.5	74–128	0.24	0.81	0.07
PIQ^b^	104.5	18.7	72–136	102.4	16.2	74–127	0.40	0.69	0.12
FIQ^c^	106.0	17.4	74–135	104.4	16.6	73–127	0.31	0.76	0.09
CTT 1^d^	99.6	17.1	61–124	86.8	19.0	55–121	2.31	0.03	0.71
CTT 2^d^	106.5	18.1	55–132	97.2	14.5	59–118	1.86	0.07	0.57
ADOS Com^e^				2.4	1.5	0–5	–	–	–
ADOS RSI^e^				6.2	3.4	1–13	–	–	–
ADOS Total^e^				8.6	3.9	3–17	–	–	–
AQ^f^	14.7	5.2	6–23	32.1	6.4	18–45	9.74	<0.001	2.98

^a^Verbal IQ (WAIS-III^UK^)

^b^Performance IQ (WAIS-III^UK^)

^c^Full-scale IQ (WAIS-III^UK^)

^d^Colour Trails Test

^e^Communication (Com), Reciprocal Social Interaction (RSI) and Total algorithm scores of the Autism Diagnostic Observation Schedule

^f^Autism-Spectrum Quotient

### Materials

Scores on the *Colour Trails Test* (CTT; D’Elia et al. 1996) were available for all participants on the database and served as the measure of executive function in this study. The CTT requires participants to connect a sequence of 25 numbers either in simple ascending order (CTT 1) or in ascending order whilst alternating the colours (pink/yellow) of the circles within which the numbers are printed (CTT 2). Completion times are standardised against age appropriate norms that are calibrated to a person’s years of education. Consistently high correlations with perseveration on the Wisconsin Card Sorting Test (WCST; Kortte et al. 2002) suggest that cognitive flexibility is among the key executive functions indexed by part 2 of the CTT whilst part 1 primarily indexes less specific perceptual-motor skills. As shown in Table 1, and in line with the literature (e.g., Hill 2004) the ASD group performed worse on both parts of the CTT than the TD group with moderate (CTT 2) to large (CTT 1) effect sizes. Since previous evidence implicates cognitive flexibility as a key executive function associated with memory difficulties in ASD (Bennetto et al. 1996; Goddard et al. 2014; Maister et al. 2013), the focus will lie on CTT 2 in the analyses reported below.

**Fig. 1:**
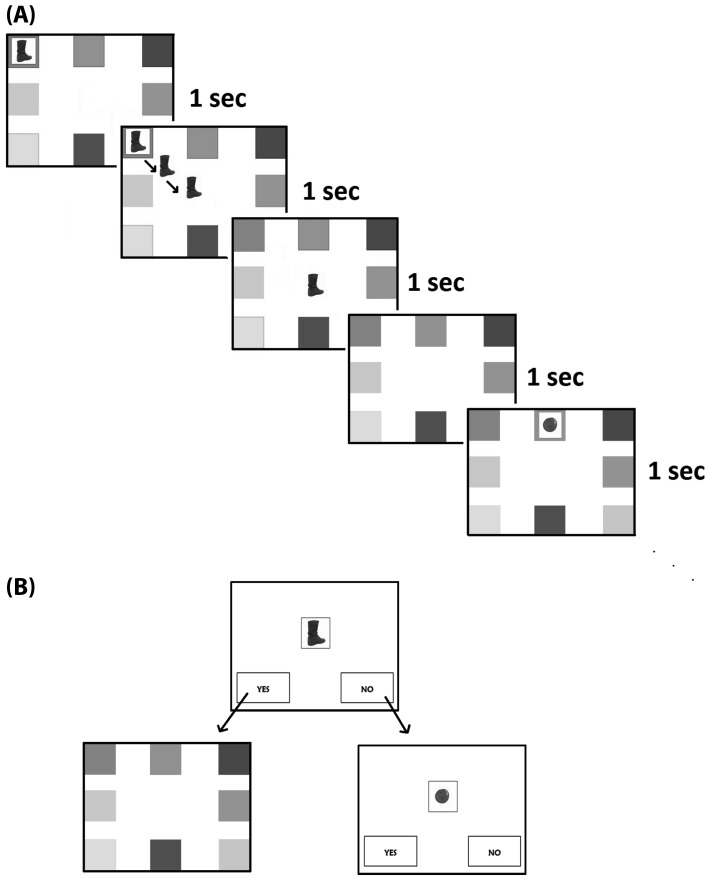
Overview of the experimental procedures. **a** illustrates the sequence of events during the study phase of an 8-location condition. For a 4-location condition only the corner locations were shown. **b** Summarises the recognition test procedure. Participants were first asked to decide if they had (yes) or had not (no) seen an object in the study phase. If they responded with ‘no’, the next object was shown. Otherwise they were asked to choose the location in which the object had been presented. (Color figure online)

For the source memory task, 150 pictures of everyday objects, taken from a standardised database (Brodeur et al. 2010), served as stimuli. Six pictures were selected for a practice trial. The remaining 144 pictures were randomly allocated to four different experimental conditions, which varied in terms of the number of objects that were to be remembered (16 or 32) and the number of source locations in which these objects were to be presented (4 or 8). Three sets of 16 objects and three sets of 32 objects were generated whilst ensuring that objects belonging to different categories (e.g., fruit) were distributed evenly across sets. The three sets of each size were rotated across participants so that all objects served equally often as the target and lure objects in the 4 and 8 location conditions. To give an example, participant 1 might study object set A in the 4-location condition, set B in the 8-location condition whilst half of set C served as the lure items during the respective recognition tests. Participant 2 might then study set B in the 4-location condition, set C in the 8-location condition with set A serving as the lures during the recognition tests; and so on. To represent the different source locations, coloured squares (e.g. red, green, yellow) were displayed in either the 4 corners of the screen (4-location condition) or the 4 corners and the four half-way points between the corners (8-location condition) as shown in Fig. 1.

### Procedure

Participants were tested individually in dedicated laboratory space in the host institution. The source memory task, which lasted approximately 45 min, was administered as part of a 2 h testing session during which participants completed also standardised clinical/neuropsychological assessments (e.g., the AQ) if not already on file, or unrelated experimental tasks (e.g., emotion-recognition). The specific combination of assessments differed across individuals but not systematically between groups and all individuals were encouraged to take breaks between assessments to avoid fatigue. During the source memory experiment described here, participants took only brief (<5 min) breaks between the four experimental conditions.

The source memory task was modelled on the computerised experiment by Russell and Jarrold (1999) and was implemented in E-Prime 2.0. Participants began with a practice task to familiarise them with the procedure. They were presented with four differently coloured locations in the 4 corners of the screen. Starting with the top left location, four objects were presented in clockwise order. Each object remained in its starting location for 1 s, then moved from there to the centre of the screen (the animation lasted 1 s) where it remained for another second. The next object was presented following a 1 s interval during which only the coloured locations were visible. Participants were instructed to remember the objects and the source locations as well as they could. After all objects were presented, participants needed to count backwards for 1 min in steps of three from 660. This distracter task was included after initial pilot testing indicated that performance may otherwise be subject to ceiling effects. Immediately following the distracter task, participants completed a memory test in which studied and lure objects were presented one at a time in random order in the centre of the screen. Two decision buttons (‘Yes’ and ‘No’) appeared underneath the object picture for participants to indicate whether or not the object had been presented earlier. If participants chose ‘No’ the next object was presented. If they chose ‘Yes’, the original source locations appeared and participants were instructed to click on the location in which the object (still seen in the centre of the screen) originally appeared. If they could not remember, they were asked to guess. Following the practice trials participants completed the four different experimental conditions in counterbalanced order, with short (1–2 min) rest periods in between. The procedure for the experimental conditions was identical to that of the practice trials but now either 16 or 32 objects appeared in either 4 or 8 locations on the screen. Figure 1 provides an overview of a sample study and test trial.

### Analysis

Two principal dependent variables were computed for each participant and for each condition to quantify performance on the source memory task. First, a corrected object recognition score was derived by subtracting the proportion of false alarms (yes responses to lure objects) from the proportion of hits (yes responses to studied objects). Second, the proportion of times participants selected the correct coloured location out of all the times they correctly recognised an object served as the source recognition score. The effects of increasing task demands and group on both measures were examined through 2 (Group; ASD vs. TD) × 2 (Objects; 16 vs. 32) × 2 (Locations; 4 vs. 8) repeated measures ANOVAs and t-tests were used to resolve significant interactions where necessary. Pearson’s correlations and step-wise regressions were used to examine associations among dependent variables. An alpha value of lower than 0.05 was considered to indicate significant effects and partial Eta squared (η_p_^2^) and Cohen’s *d* are reported throughout as estimates of effect sizes with 90 and 95% confidence intervals respectively (see Steiger 2004).

## Results

### Object Recognition

Table 2 sets out the object recognition data. A 2 (Group) × 2 (Objects) × 2 (Locations) ANOVA of the corrected recognition rates demonstrated a significant main effect of the number of objects, *F*(1,41) = 7.49, *p* = 0.009, η_p_^2^ = 0.15, 90% CI (0.02–0.31), with overall higher performance on conditions with 16 (M = 0.77; SD = 19) compared to 32 objects (M = 0.73; SD = 0.19). The effect of the number of source locations was not significant, *F*(1,41) = 0.34, *p* = 0.56, η_p_^2^ < 0.01, 90% CI (0.00–0.10), suggesting that increasing the number of source locations has minimal effects on item memory. Unexpectedly, there was also a main effect of group, *F*(1,41) = 7.77, *p* = 0.008, η_p_^2^ = 0.16, 90% CI (0.03–0.32), with higher corrected object recognition for the TD (M = 0.82; SD = 0.14) compared to the ASD group (M = 0.68; SD = 0.20) across all conditions. None of the interactions was significant (Max *F* = 0.74, min *p* = .40, max η_p_^2^ = 0.02).

**Table 2:** Hit rates, false alarm rates and corrected recognition rates (hits minus false alarms) for the ASD and TD groups’ object recognition as a function of task demands

Measure	Object × location	TD		ASD		Cohen’s *d*
		M	SD	M	SD	
Hits	4 × 16	0.88	0.15	0.78	0.18	0.60
	4 × 32	0.84	0.14	0.74	0.22	0.54
	8 × 16	0.87	0.14	0.77	0.16	0.67
	8 × 32	0.84	0.13	0.73	0.18	0.70
False Alarms (FA)	4 × 16	0.03	0.04	0.07	0.11	0.48
	4 × 32	0.04	0.04	0.06	0.08	0.32
	8 × 16	0.01	0.03	0.08	0.13	0.74
	8 × 32	0.04	0.07	0.09	0.12	0.51
Hits—FA	4 × 16	0.84	0.17	0.70	0.23	0.69
	4 × 32	0.80	0.15	0.68	0.23	0.62
	8 × 16	0.86	0.16	0.69	0.21	0.91
	8 × 32	0.79	0.17	0.64	0.24	0.72

To further clarify the main effect of group, separate ANOVAs on the hit and false alarm rates were carried out, which indicated that the ASD group achieved significantly fewer hits, *F*(1,41) = 5.18, *p* = 0.028, η_p_^2^ = 0.11, 90% CI (0.01–0.27) and committed a greater number of false alarms, *F*(1,41) = 3.99, *p* = 0.0052, η_p_^2^ = 0.09, 90% CI (0.00–0.24). This observation renders it unlikely that the groups adopted very different response criteria since a more liberal criterion would have resulted in greater hits *and* greater false alarms whereas a more conservative criterion would result in fewer hits *and* fewer false alarms.

### Source Memory

**Fig. 2:**
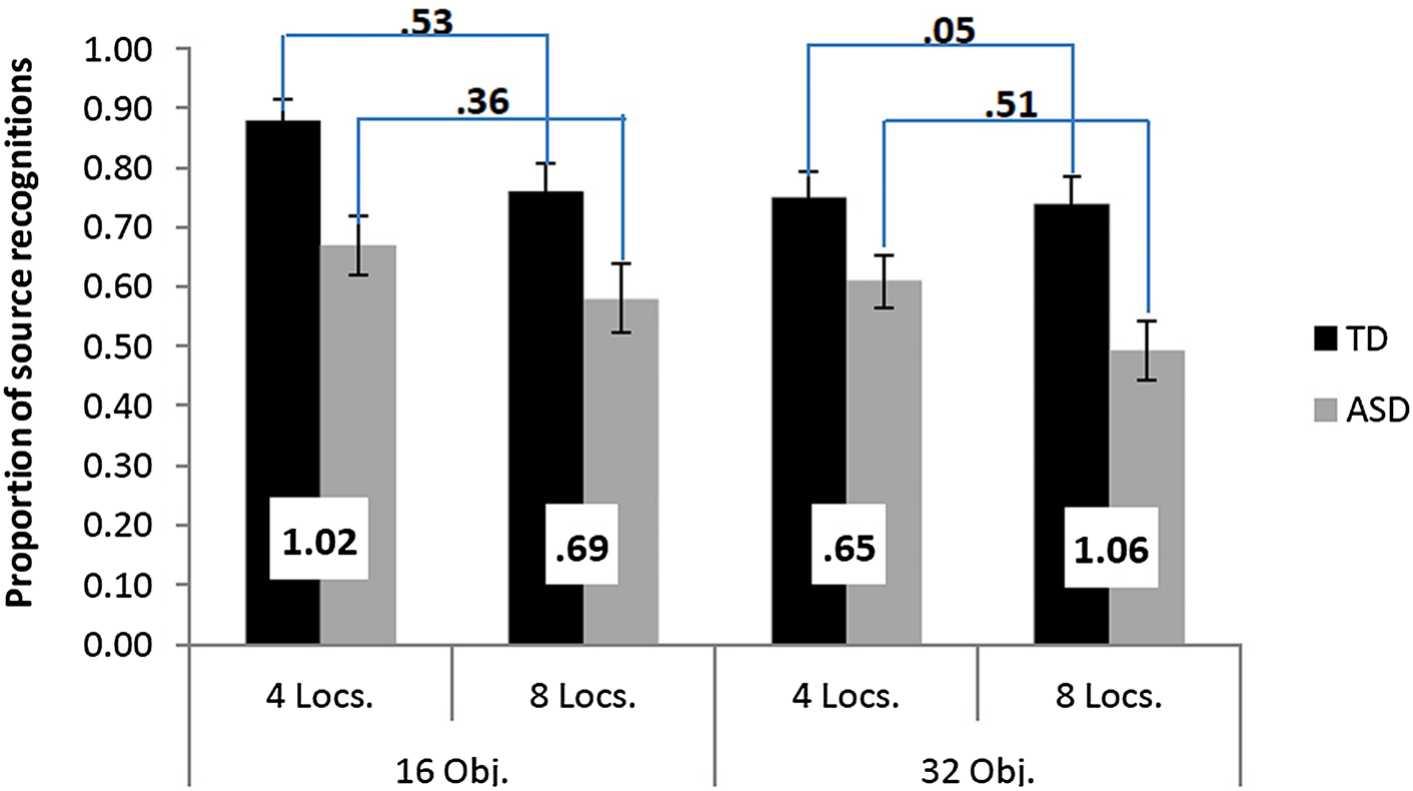
Proportion of correctly identified source locations for the ASD (grey) and TD (black) groups as a function of the number of source locations (4 vs. 8 Locs.) and objects (16 vs. 32 Obj.). Error bars represent +/− 1 SE and the inset values represent Cohen’s *d* effect sizes for the relevant between group and within group comparisons

The source memory data are set out in Fig. 2. A 2 (Group) × 2 (Objects) × 2 (Locations) repeated measures ANOVA revealed significant main effects of the number of source locations, *F*(1,41) = 15.26, *p* <  0.001, η_p_^2^ = 0.27, 90% CI (0.09–0.43), and objects, *F*(1,41) = 9.26, *p* = 0.004, η_p_^2^ = 0.18, 90% CI (0.04–0.35), with higher source recognition rates for the 4 (M = 0.72; SD = 0.20) compared to the 8 location (M = 0.64; SD = 0.25) condition, and higher performance for the 16 object (M = 0.72; SD = 0.23) compared to 32 object (M = 0.65; SD = 0.23) condition. Together with the lack of an interaction between the object and location factors, *F*(1,41) = 1.26, *p* = 0.27, η_p_^2^ =  0.03, 90% CI (0.00–0.15), these main effects indicate that increasing the number of to-be-remembered objects leads to similar source memory decrements as increasing the number of source locations.

The ANOVA also confirmed a significant group effect, *F*(1,41) = 10.41, *p* = 0.002, η_p_^2^ = 0.20; 90% CI (0.05–0.36), with lower source memory scores in the ASD (M = 0.59; SD = 0.21) than in the comparison group (M = 0.78; SD = 0.19). Of most interest, however, was that whilst neither the group x locations, *F*(1,41) =  1.48, *p* =  .23, η_p_^2^ =  0.04, 90% CI (0.00–0.16), or group x objects, *F*(1,41) =  0.00, *p* =  .97, η_p_^2^ =  0.001, 90% CI (0.00–0.00) interactions were significant, there was a significant three-way interaction, *F*(1,41) =  4.59, *p* =  0.038, η_p_^2^ =  0.10, 90% CI (0.00–0.25), which is best understood with reference to Fig. 2. Considering first only the data from the 16 object condition, it can be seen that the main effects of the number of locations, *F*(1,41) =  12.36, *p* =  0.001, η_p_^2^ =  0.23, 90% CI (0.06–0.39) and group, *F*(1,41) =  8.95, *p* =  0.005, η_p_^2^ =  0.18; 90% CI (0.03–0.34) were not characterised by an additional interaction, *F*(1,41) =  0.71, *p* =  .79, η_p_^2^ =  0.002, 90% CI (0.00–0.13), indicating that ASD and TD participants demonstrated similar source memory decrements when a relatively small number of items needed to be encoded under increasing numbers of contextual conditions. By contrast, for the 32 object condition the main effects of location, *F*(1,41) =  7.48, *p* =  0.018, η_p_^2^ =  0.15, 90% CI (0.02–0.31) and group, *F*(1,41) =  9.08, *p* =  0.004, η_p_^2^ =  0.18, 90% CI (0.04–.34), were characterised by an interaction [*F*(1,41) =  6.13, *p* =  0.018, η_p_^2^ =  0.13, 90% CI (0.01–0.29)] whereby the ASD group demonstrated a more pronounced source memory difficulty vis-a-vis the comparison group in the 8 location (Cohen’s *d* =  1.07, 95% CI 0.53–1.60) versus the 4 location condition (Cohen’s *d* =  0.62, 95% CI 0.10–1.1). Although this pattern is partly in line with predictions, it is worth noting that there were also substantial group differences in what should be the least demanding condition (16 items in 4 locations), where the TD group demonstrated their best source memory scores whilst the ASD group’s performance was in line with their performance on the other conditions (see Fig. 2).

### Correlations Between Measures and Between Conditions

Table 3 sets out the correlations between the memory measures and participants’ verbal abilities (VIQ) and executive functions (CTT 2). The pattern across both groups generally confirms earlier reports of associations between memory and verbal as well as executive abilities (e.g., Bennetto et al. 1996; Hala et al. 2005; Maister et al. 2013). Within groups, however, the correlations between the CTT 2 and object recognition and between CTT 2 and source memory were only significant in the TD but not the ASD group. Correlations between VIQ and the memory measures, on the other hand, were significant in both groups. Of interest also was the very robust correlation between object recognition and source memory in both groups, which could shed some light on the unexpected observation of an object recognition decrement in the earlier analysis. Specifically, it is possible that the ability to remember the source locations of objects confers advantages also for recognising the objects per se, which would put individuals with ASD at a disadvantage because of their source memory difficulties. To address this possibility and further clarify the role of verbal ability and executive functions in the memory difficulties associated with ASD, two step-wise regressions were carried out.

**Table 3:** Correlations between object recognition, source memory, participant’s verbal ability and executive functions across both groups and within the ASD and TD groups alone

Groups	Variable	Object Rec.	Source memory	VIQ
Combined	Source memory	0.77**		
	VIQ	0.52**	0.52**	
	CTT2	0.52**	0.47**	0.46**
TD only	Source memory	0.85**		
	VIQ	0.64**	0.69**	
	CTT2	0.60*	0.64**	0.64**
ASD only	Source memory	0.66**		
	VIQ	0.51*	0.46*	
	CTT2	0.22	0.15	0.29

**p < 0.005; *p < 0.05

In the first analysis, object recognition served as the dependent variable and group, VIQ and CTT 2 and source memory were successively added as predictors. As the top half of Table 4 shows, group (β = 0.34, *p* = 0.009) and VIQ (β = 0.35, *p* = 0.022) were both significant and independent predictors of object recognition when these were added along with CTT 2 as the only predictors in the model. However, when source memory was added, this factor alone predicted object recognition (β =  0.60, *p* < 0.001) and neither group nor VIQ accounted for any additional variance. By contrast, when object recognition was added after group, VIQ and CTT 2 had been added as predictors of source memory, group (β =  0.20, *p* = 0.080) remained a marginally significant predictor along with object recognition (β =  0.57, *p* <  0.001). In other words whilst individual differences in source memory explained much of the group difference in object recognition, individual differences in object recognition did not seem to fully account for the group differences in source memory. Executive abilities as indexed by the CTT 2 did not, independently, explain a significant portion of the variance in the memory measures of any of the models.

**Table 4:** Hierarchical regression examining the predictors of object recognition and source recognition

Dependent	Model (adjusted R^2^; F test)	Predictors	Beta	*t*	*p*
Object Rec	Step 1: R^2^ = 0.16 (*F*(1,41) = 8.62; *p* = 0.005)	Group	0.42*	2.94	0.005
	Step 2: R^2^ = 0.41 (*F*(3,39) = 10.45; *p* < 0.001)	Group	0.34*	2.73	0.009
		VIQ	0.35*	2.38	0.022
		CTT 2	0.24	1.59	0.121
	Step 3: R^2^ = 0.60 (*F*(4,38) = 16.33; *p* < .001)	Group	0.11	0.96	0.344
		VIQ	0.12	0.92	0.366
		CTT 2	0.12	0.96	0.342
		Source Rec	0.60*	4.37	<0.001
Source Rec	Step 1: R^2^ = 0.18 (*F*(1,41) = 10.41; *p* = 0.002)	Group	0.45*	3.23	0.002
	Step 2: R^2^ = 0.43 (*F*(3,39) = 11.73; *p* < 0.001)	Group	0.39*	3.19	0.003
		VIQ	0.43*	3.27	0.002
		CTT 2	0.16	1.18	0.245
	Step 3: R^2^ = 0.62 (*F*(4,38) = 18.14; *p* < 0.001)	Group	0.20	1.80	0.080
		VIQ	0.18	1.49	0.145
		CTT 2	0.08	0.71	0.483
		Object Rec	0.57*	4.49	<0.001

*Identifies the significant predictors in each model

## Discussion

Motivated by inconsistencies in the literature, the current study set out to examine the role of task demands and individual differences in executive functions and verbal ability in source memory in ASD. Previously, Bowler et al. (2004, 2015) had shown that source memory difficulties in ASD are moderated by whether participants are asked to recall contextual source information or identify the same information from a list of options. Since that observation did not resolve all inconsistencies in the literature, the current study examined the possible influences of additional task factors by systematically varying the number of to-be-remembered items and the number of to-be-remembered source locations. Our prediction was that increases in the number of source locations would disproportionately affect source memory in ASD because of the increasing demands on associating specific locations with the items. By contrast, increasing the number of items was thought to have more similar effects on both groups since this would primarily increase the demands on item memory, which is generally thought to be preserved in ASD.

The results were partly in line with predictions. First, the observations confirmed that both groups demonstrated poorer source memory when 32 instead of 16 objects needed to be remembered and when these were presented in 8 instead of only 4 screen locations. The results also confirmed that increasing the number of items had similar consequences for source memory in ASD and comparison groups whilst increases in the number of source locations had more detrimental effects for individuals with ASD, albeit in only the condition where a relatively large number of items needed to be studied. Finally, the results across both groups confirmed previous reports of associations between memory and participants’ verbal and executive abilities although within both groups only verbal ability was significantly associated with memory whilst a measure of executive function was associated with memory only in the TD but not the ASD group. Before we consider the implications of these findings for the source memory literature, we will first turn to the implications of the unexpected object recognition decrement in ASD that was contrary to predictions.

The observation of a recognition decrement for single items is not unique in the autism literature, however, a comprehensive review by Boucher et al. (2012) concluded that such difficulties are normally only observed in individuals with ASD who demonstrate additional intellectual impairments, or when memory is assessed for face stimuli. There are some noteworthy additional exceptions, however, on which the current study sheds some new light. Specifically, a number of studies have found poorer recognition memory in ASD specifically for words that are encoded self-referentially rather than with reference to their physical properties (Henderson et al. 2009; Lombardo et al. 2007; Toichi et al. 2002). This finding is typically interpreted as a reflection of abnormalities in aspects of self-referential thinking rather than memory. Some studies have also observed recognition difficulties for individual objects embedded in complex scenes (Cooper et al. 2015; Ring et al. 2015) or for objects that are presented simultaneously alongside other objects during study (Bennetto et al. 1996; Bowler et al. 2014; Ring et al. 2016). Finally, and most directly related to the current study, Bowler et al. (2004) observed object recognition impairments in the context of their source memory study that required participants to remember either who, where or how different words were presented. All of these studies have in common that to-be-remembered items were encoded in relation to specific contextual details that participants either encoded intentionally or incidentally. The current study demonstrates that the ability to remember specific contextual details is strongly associated with single-item recognition and that decrements in source memory can account for much of the object recognition difficulties in ASD. By contrast attenuated object recognition cannot fully explain source memory difficulties. This asymmetrical relationship between memory for single items and the contexts within which they are studied suggests that the difficulties individuals with ASD experience in the encoding and recollection of contextually rich representations of events can have consequences for their ability to recognise individual event details, which has important practical implications.

Specifically, the observation suggests that certain memory difficulties in ASD should be alleviated by re-instating as much of the original study context as possible. In educational and intervention settings, for instance, it may help children to generalise what they learn at school or in therapy to the home environment (and vice versa) if certain contextual details (e.g., room decorations and materials used during learning) are replicated in both settings and only gradually varied. Studies of eye-witness testimony already lend some support to this suggestion by showing that bringing participants back into the environment in which they had watched a recorded crime, alleviates a difficulty individuals with ASD normally experience in retrieving important details about the witnessed event (Maras and Bowler 2012a). Interestingly, however, only the physical reinstatement of the study context is effective in these circumstances whereas instructing participants to mentally bring to mind contextual details about the study environment is of little benefit. The current study helps to explain this pattern by suggesting that difficulties in spontaneously retrieving contextual information may be among the reasons why individuals with ASD find it hard to retrieve certain event details in the first place. Thus, the current findings help to further specify the conditions under which task support can effectively alleviate some of the memory difficulties that individuals with ASD experience (see also Gaigg and Bowler 2012). From a more theoretical standpoint, the observations also call attention to the fact that the processes involved in the encoding and retrieval of single items do not operate independently from those involved in the encoding and retrieval of the associated contexts, both in comparison and ASD individuals.

Turning to the source memory data, the findings were only partly in line with predictions. Specifically, the data confirmed that increasing the demands on item memory had similar consequences for source memory in ASD and TD participants. However, increasing demands on associative processes by varying the number of source locations from which the to-be-remembered items originated had more detrimental effects for individuals with ASD only when a relatively large but not a small number of items needed to be studied. With hindsight, this pattern is perhaps not that surprising given that the data more generally showed that item and source memory operate less independently than might be expected. Although the results, therefore, lend some support to the suggestion that certain task demands moderate source memory difficulties in ASD, the results do not resolve remaining inconsistencies in the literature because individuals with ASD demonstrated fairly pronounced source memory difficulties under all experimental conditions. Interesting, in this context, is that the only pair of previous studies that have used nearly identical procedures, similar to those employed here, have yielded entirely opposite results. Specifically, Russell and Jarrold (1999) as well as Williams and Happé (2009) asked children with and without ASD to remember whether they or the experimenter placed picture cards on a game-board on their own behalf or that of a toy partner. At test, participants were asked to hand the pictures back to the person or toy partner who had placed them. Russell and Jarrold (1999) reported very substantial source memory impairments in their group of children with ASD, whereas Williams and Happé (2009) reported none. The only substantial differences between these studies was that the latter involved children with less severe intellectual impairments than the former and therefore Williams and Happé (2009) used 32 instead of 24 object cards to avoid potential ceiling effects in performance. If anything, the current study suggests that using 32 instead of only 24 objects should have made it more likely for Williams and Happé (2009) to replicate the source memory difficulties observed by Russell and Jarrold (1999). Moreover, as the authors note, differences in the developmental level of the participants is an unlikely reason for the discrepant findings given that many other studies have demonstrated source memory difficulties in children with no intellectual impairments at all (the current study represents another example).

An interesting suggestion by Williams and Happé (2009) in relation to these inconsistent findings was that the children in their study may have engaged more elaborative encoding strategies than the children in Russell and Jarrold (1999) because of a strongly encouraged verbal commentary about the ongoing picture placements. Although only speculative, this suggestion is in line with other observations which suggest that supporting certain encoding processes through explicit instructions often leads to preserved memory in ASD even when free recall procedures are used (Mottron et al. 2001; Toichi and Kamio 2002; but see; Smith et al. 2007). In the current study, no specific encoding instructions were given but it is possible that comparison participants adopted different strategies for the 16 and 32 item conditions whilst ASD participants engaged a less flexible strategy across all conditions. This possibility would be in line with the three-way interaction (see Fig. 2), and also with the more pronounced correlations between memory measures and CTT 2 in the comparison than the ASD group. As noted earlier, the CTT 2 is thought to index cognitive flexibility (Kortte et al. 2002) and the impairments in this domain in ASD (see Table 1) might lead them to rely on less flexible verbal mediation strategies than the comparison group or they may have fewer executive functions to rely on in the more demanding condition. Although speculative, this interpretation could be empirically tested in future studies through manipulations that encourage only particular encoding strategies (e.g., item specific vs. relational strategies) thereby limiting any advantage that TD participants might have in flexibly adapting to varying task demands.

There are a number of limitations that need to be acknowledged. First, an important caveat to the conclusions just outlined, is that we only had a single measure of executive function available to inform the possible role of executive functions in the source memory difficulties in ASD. Although widely used in the neuropsychological literature, it captures only some of the processes thought to constitute the construct of executive functions and future studies should follow the example of recent studies by Goddard et al. (2014) and Maister et al. (2013) to examine executive functions through a broader battery of executive function measures. Interesting, in this context, however is that both of those studies included a measure of cognitive flexibility and both reported strong associations between this measure and memory impairments in ASD. It is unlikely that our failure to replicate this observation is simply the result of our relatively modest sample size because Maister et al. (2013) had sample sizes of only 14 participants in each group and our sample is large enough to detect other expected correlations of the same magnitude (i.e., associations with verbal ability). A more likely reason is that our sample included adults rather than children. Although there were no correlations between age and any other dependent variables in the current study, adults with ASD may rely less on their cognitive flexibility to meet certain task demands than children, or there might be more pronounced individual differences in this respect in adulthood than childhood. Another potential limitation of the current study is that item and source memory were probed in the same test procedure, potentially inflating the association between item and source memory. It would be useful for future studies to examine associations between item and source memory across different experimental procedures.

 Notwithstanding certain caveats, we believe that the current observations make an important contribution to the memory literature in ASD by shedding light on task parameters that moderate source memory difficulties in ASD and that may therefore contribute to inconsistent findings in this literature. The findings are also important because they suggest that the difficulties individuals with ASD experience in remembering contextual information can have consequences for their ability to remember single items. The findings, therefore, have important practical implications by further specifying the conditions under which task support might be effectively used to alleviate memory difficulties in ASD and also theoretical implications by highlighting that the processes that serve item and source memory often interact, or are, in fact, the same.

## Footnotes

1 In their second of two experiments, Bigham et al. 2010 presented children with a series of 10 shapes and asked them to perform a unique manual action for each one. During test children were presented with the shapes and asked to reproduce the actions. Although Bigham et al. 2010 describe this as a source memory paradigm, the fact that each shape was associated with a unique action also satisfies the definition of a paired associate cued recall test.
